# Optimization of mucilage secretion of *Plantago ovata, Alyssum desertorum*, and *Ocimum basilicum* seeds by response surface method

**DOI:** 10.1186/s13104-023-06517-6

**Published:** 2025-04-22

**Authors:** Bahram Tafaghodinia

**Affiliations:** Department of plant production, Agricultural Institute, Iranian Research Organization for Science and Technology, Tehran, Iran

**Keywords:** Mucilage secretion, *Plantago ovata*, *Alyssum desertorum*, *Ocimum basilicum*, Response surface methodology (RSM), Seed mucilage optimization

## Abstract

Diaspore comprises a collection of seed coats and other organ components that effectively protect the embryo and dispersal. The mucilage in many species, a gelatinous mixture of cell wall polysaccharides, is secreted by the pericarp. Although the modification, synthesis, and secretion of the strain of mucilage as well as structure and roles of plant cell wall have been of great interest and study by researchers, the understanding of the best conditions for the secretion of mucilage has received low attention. This research aimed to investigate effective factors in the process of mucilage secretion in *P. ovata, A. desertorum*, and *O. basilicum* seeds. To achieve this objective, an investigation was conducted to examine the impact of seed number, polarity, pH and species on mucilage secretion. This study showed that the number of seeds and type of species had the least and the most effect on mucilage production, respectively. Finally, according to the results of Response Surface Method design experiments, pH, polarity and species had a significant effect on the process of mucilage secretion. pH = 4 and polarity = 15 for *P. ovata* and pH = 10 and polarity = 15 for *O. basilicum* and *A. desertorum* were the most favorable conditions for secretion of 10.3 mm, 9.4 mm, and 2.9 mm of mucilage, respectively.

## Introduction

The pericarp, which forms the outer layer of the seed, plays a crucial role in seed protection and development by producing mucilage [1]. Mucilage is a viscous, sticky substance that is produced by plants and is composed of polysaccharides, which are essentially long chains of sugars [2, 3]. By covering the surface of a seed, mucilage can trap moisture and inhibit its rapid evaporation. This feature is particularly critical for plants that grow in dry or arid environments [4, 5].

Extensive research has been carried out to optimize the secretion process of mucilage from various plant sources [5–10]. In one study, researchers explored the impact of various secretion parameters, such as temperature, NaCl concentrations, and pH, on the germination and metabolism from *A. alpinus* subsp. The study concluded that the ideal secretion conditions are a temperature of 15 °C, pH = 7 and low concentration of NaCl [11]. In 2020, researchers studied the effect of pH, temperature, and secretion time on the yield and rheological properties of mucilage extracted from fenugreek seeds. The study found that the optimal secretion conditions were a pH of 8, a temperature of 60 °C, and an secretion time of 3 h [8]. In a study published in 2023, researchers investigated the effect of different secretion solvents (including ethanol and isopropanol) on the yield and functional properties of mucilage extracted from *Opuntia ficus-indica* (Cactaceae). The study found that isopropanol was the most effective solvent for mucilage secretion [12]. In 2022, a group of researchers conducted a study to examine the influence of solvent concentration, temperature, and secretion time on the yield and rheological properties of mucilage extracted from Indian tamarind seeds. The study demonstrated that the optimal secretion conditions were achieved with a solvent concentration of 1%, an secretion temperature of 70 °C, and an secretion time of 5 h [10]. Similarly, a study published in 2022 aimed to evaluate the effect of varying secretion conditions, such as solvent concentration, temperature, and secretion time, on the yield and functional properties of mucilage extracted from flaxseed hulls. The findings revealed that the most favorable secretion conditions were a solvent concentration of 3%, a temperature of 60 °C, and an secretion time of 2 h [6]. In another study to determine the optimal conditions for extracting mucilage from whole flaxseed, the authors found that the maximum yield of flaxseed mucilage was obtained at 80 ± 2 °C with a seed to water ratio of 1 to 25, without the need for swelling [13].

This study aimed to evaluate the impact of polarity (average polarity), pH, and seed number on the level of mucilage secretion from *Plantago ovata, Alyssum desertorum*, and *Ocimum basilicum* seeds using the Response Surface Method (RSM). The main objective was to determine the optimal conditions for mucilage secretion, with a focus on investigating their respective water holding capacity conditions.

## Experimental investigation

### Secretion mucilage optimization

To examine the combined impact of pH, polarity, number, and seed type on mucilage secretion, experiments were conducted using a response surface method. The temperature (27 ± 2) and light (light 16 h and darkness 8 h) were kept constant, and the details of the variables are outlined in Table 1. Table 2 presents the key characteristics of the dependent variable mucilage secretion diameter. Seeds from *P. ovata, A. desertorum*, and *O. basilicum* were procured from the Seed and Plant Improvement Institute (SPII), and the number of seeds varied across six levels ranging from 3 to 8. The ethanol polarity effect was tested by varying the concentration across five levels (5–50%), Meanwhile, the impact of pH on gelation was examined by utilizing various pH solutions spanning 5 levels, ranging from 1 to 13. trypan blue, Solvents, and Congo red dyes were attained from Sigma Company and used to stain the samples, and the pH of the solutions was measured using a digital pH meter after preparing them with HCl and NaOH. The behavior of each treatment was monitored for 30 min using a camera, and after 24 h, the mucilage diameter secretion and the measurement of the average distance between the grains was conducted. A camera was used to obtain a more accurate time of maximum mucilage secretion. The seeds were gelled by assessing the treatments inside a six-well microplate on a weighing table to remove vibration during capturing.

**Table 1:** Variables (pH, Polarity, Seed number and species) and response related to mucilage secretion by Response Surface Method design experiment

Factors	Name	Type	Minimum	Maximum	Mean	SD.
X_1_	pH	Num.	1	13	7	2.70
X_2_	Polarity	Num.	5	45	25	9.01
X_3_	number	Num.	3	7	5	0.90
X_4_	Species	Cat.	*O. basilicum*	*A. desertorum*	Levels	3

**Table 2:** RSM design experiment for 4 independent variables: X_1_:pH, X_2_:Polarity, X_3_:Seed number and X_4_: Species

Response	Name	Units	Minimum	Maximum	Mean	SD
R1	mucilage	mm	0	10.6	4.3	3.2

### Capacity of water retention

To investigate the mucilage water retention ability and seeds after drying, ten seeds from each group were weighed in triplicate, immersed in distilled water for 30 min, and weighed. The seeds were placed on a scale accurate to 0.0001 units, and their weights were recorded every 5 min for a duration of 200 min until they were dehydrated at laboratory temperature (20 °C).

### Design experiments

This study employed a response surface method (RSM) to design and implement 60 experiments (treatments and repetitions). The experiments were designed and the results were analyzed using Design Expert (Ver.13) and Qualitek- 4 (student Ver.) software, which was also used to generate relevant graphs.

## Result and discussion

Table 2 displays the results of the experimental design and their corresponding matrix. Table 3 presents the ANOVA for a response surface quadratic model for mucilage secretion. The RSM analysis indicated that all four variables had a positive impact on the mucilage secretion effectiveness (Y). Model terms were regarded as statistically significant if the “probability > F” values were below 0.05. The analysis of ANOVA revealed that the main effects of all variables were significant. The main effects of seed type and polarity showed the most significant impact (P-value < 0.0001). Furthermore, most variable interactions were found to be statistically significant based on the P-values of the interaction terms, as shown in Table 3.

**Table 3:** The analysis regression for secretion of mucilage from *P. ovata, A. desertorum*, and *O. basilicum* seeds for quadratic model fitting (ANOVA)

Run	X_1_:pH	X_2_:Polarity	X_3_:Seed number	X_4_: Species	Mucilage secretion (0. 1 mm)
1	7	25	5	*P. ovata*	5
2 3	7	45	5	*O. basilicum*	1.4
3	1	25	5	*O. basilicum*	0.8
4	10	35	6	*O. basilicum*	0.2
5	7	25	7	*O. basilicum*	0.2
6	7	25	5	*P. ovata*	2.6
7	7	25	5	* A. desertorum*	8.5
8	4	35	4	*O. basilicum*	0
9	7	25	3	*O. basilicum*	2.1
10	10	35	6	* A. desertorum*	9.2
11	1	25	5	*P. ovata*	0.4
12	4	15	6	* A. desertorum*	7
13	10	15	6	*O. basilicum*	6
14	10	15	4	*P. ovata*	8
15	4	35	6	*O. basilicum*	10.
16	10	35	4	* A. desertorum*	2.4
17	13	25	5	* A. desertorum*	3.4
18	7	5	5	*P. ovata*	9
19	7	25	5	*O. basilicum*	1.8
20	10	35	6	*P. ovata*	0
21	10	15	4	*O. basilicum*	9.6
22	4	35	6	* A. desertorum*	6.2
23	4	15	4	*O. basilicum*	0
24	10	15	4	*A. desertorum*	6.4
25	13	25	5	*P. ovata*	5.3
26	4	35	4	* A. desertorum*	3.1
27	4	15	6	*P. ovata*	6.6
28	7	45	5	* A. desertorum*	4.6
29	7	25	5	* A. desertorum*	5
30	7	25	3	*A. desertorum*	1
31	7	25	7	*P. ovata*	5.6
32	7	25	5	*P. ovata*	4
33	7	25	5	*A. desertorum*	
34	7	25	5	*P. ovata*	0.8
35	7	25	7	* A. desertorum*	4
36	7	5	5	*A. desertorum*	2.6
37	7	25	5	*O. basilicum*	2.2
38	7	25	5	*P. ovata*	4.2
39	7	25	5	*P. ovata*	4.2
40	7	45	5	*P. ovata*	3.8
41	13	25	5	*O. basilicum*	3.8
42	1	25	5	* A. desertorum*	9.8
43 30	7	25	5	* A. desertorum*	10.6
44	4	35	4	*P. ovata*	3.2
45	10	35	4	*O. basilicum*	4
46	7	5	5	*O. basilicum*	7
47	7	25	5	*O. basilicum*	2.4
48	10	35	4	*P. ovata*	0.4
49	7	25	5	*O. basilicum*	0.4
50	10	15	6	*P. ovata*	8.4
51	7	25	3	*P. ovata*	3.6
52	7	25	5	*O. basilicum*	2.2
53	7	25	5	*O. basilicum*	2.8
54	4	35	6	*P. ovata*	9.1
55	4	15	4	* A. desertorum*	9.8
56	4	15	6	*O. basilicum*	0
57	10	15	6	*A. desertorum*	7.6
58	7	25	5	* A. desertorum*	8.8
59	4	15	4	*P. ovata*	0
60	7	25	5	*A. desertorum*	7.3

The model’s standard deviation was determined to be 1.49. This small value indicates that the model is reliable and can provide predicted values that are close to the real values of the responses. Based on the significant terms, the polynomial model for mucilage production across three seeds using the experimental data can be quantified as follows:

Production of mucilage - *P.ovata*(mm)= -24.83735+(5.26410*X_1_)+(0.223996*X_2_)+ (3.39089*X_3_) – (0.090417*X_1_ × _2_) – (0.526389*X_1_ × _3_) – (0.018750*X_2_ × _3_) – (0.015194*X_1_^2^) + (0.007466*X_2_^2^) + (0.296591*X_3_^2^).

Mucilage production - *A.desertorum* (mm) = + 4.28311 – (2.68577*X_1_) – (0.821510*X_2_) + (9.10841*X_3_) + (0.020972*X_1_ × _2_) + (0.323611*X_1_ × _3_) + (0.142917*X_2_ × _3_) + (0.035603*X_1_^2^) – (0.003129*X_2_^2^) – (1.40570*X_3_^2^).

Mucilage production - *O. basilicum* (mm) = -16.82215 + (4.28682*X_1_) – (0.320367*X_2_) + (3.39089*X_3_) – (0.064421*X_1_ × _2_) –(0.477542*X_1_ × _3_) + (0.048263*X_2_ × _3_) + (0.011935*X_1_^2^) + (0.008876*X_2_^2^) – (0.171751*X_3_^2^) where X1, X2, X3, and X4 represent pH, Polarity, number, and seed, respectively.

The model’s statistical significance was substantiated by both the Fisher F-test (showing an F-value of 7.93) and ANOVA analysis (displaying a p-value of less than 0.0001). The model’s F-value (7.93) indicates that the model is significant, with only a 0.01% chance that the F-value could occur due to noise. Moreover, the p-values less than 0.05 suggest that the model terms are significant. Specifically, the X_1_, X_2_, X_3_, X_4_, X_1_ × _2_, X_1_ × _4_, X_3_ × _4_, X_22_, X_32_, X_1_ × _2_ × _4_, X_1_ × _3_ × _4_, X_32_, and X_4_ terms were found to be significant in the model. According to these findings, the derived regression model holds significant statistical significance. A Lack of Fit F-value of 0.92 suggests its insignificance in comparison to the pure error. This suggests a good fit of the model, and there is no significant difference between predicted and actual values. he robust correlation between the predicted and experimental data was further acknowledged by the non-significant lack of fit, pure error, determination coefficient (R^2^ = 0.9020), and adjusted determination coefficient (Adj R^2^ = 0.7883). These results suggest that the regression model accurately represents the data and can be used to predict mucilage production in three seeds. Hence, the developed model is capable of elucidating 90.20% of the variation in the response through the incorporation of all independent variables (R2), and 78.83% when considering solely the influential independent variables on the dependent variable (Adj R2). Moreover, the predictive determination (Pred R2 = 0.4477) affirms the model’s strong predictive capacity for existing experimental data, while its ability to predict new observations is limited. Furthermore, the assessment of signal-to-noise ratio, specifically the adequacy precision value exceeding 4 (9.40), underscores that the established model effectively navigates the design space.

**Fig. 1:**
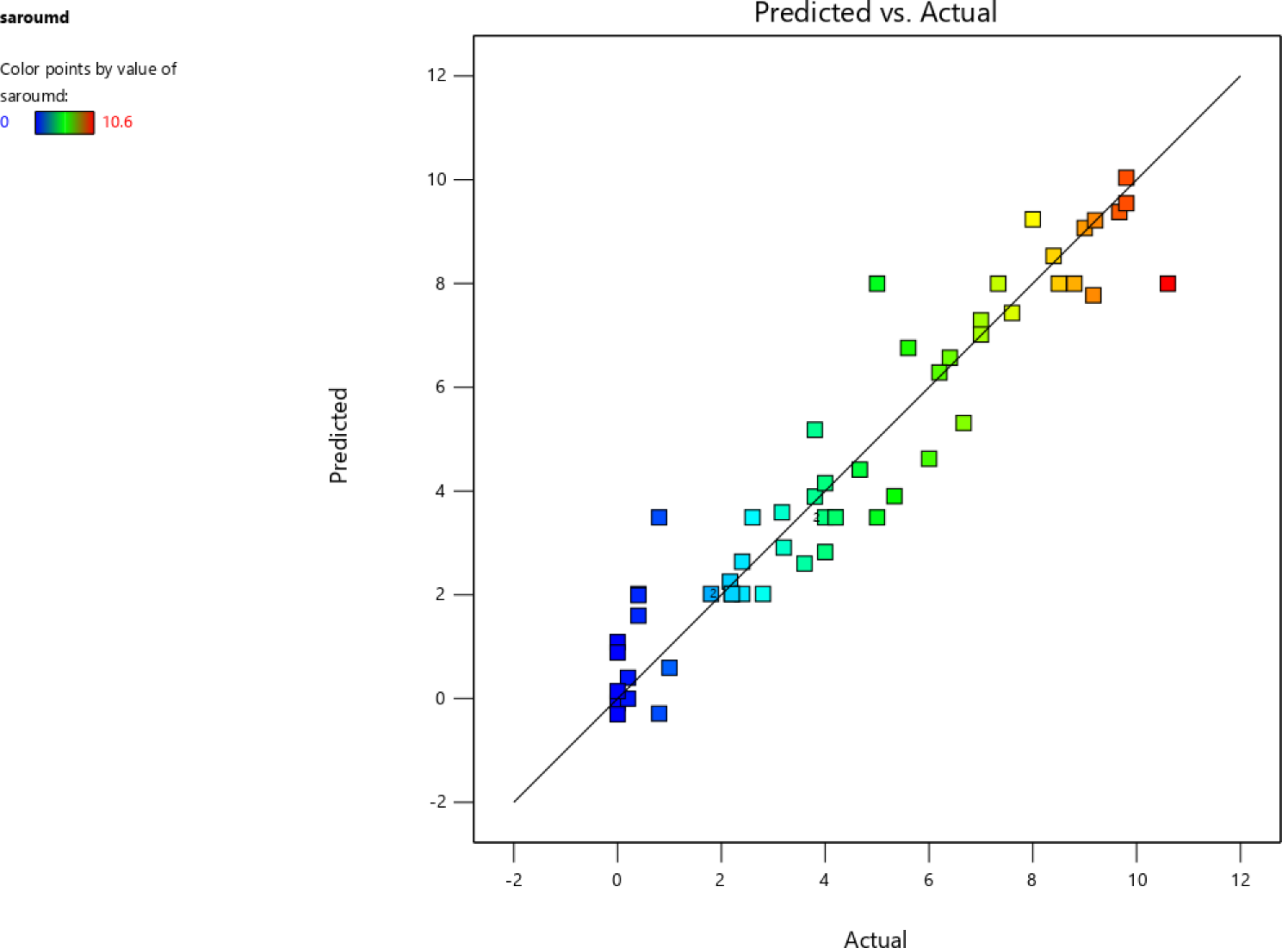
Predicted values versus externally studentized residual of the mucilage secretion of *P. ovata*, A. *desertorum*, and O. *basilicum* seed

Furthermore, Fig. 1 displays the comparison between the predicted and experimental values. This figure provides evidence of the achievement of the formulated models demonstrate a good ability to establish meaningful correlations between the variables and the corresponding responses, as it demonstrates the close proximity of the predicted values to the experimental values.

**Fig. 2:**
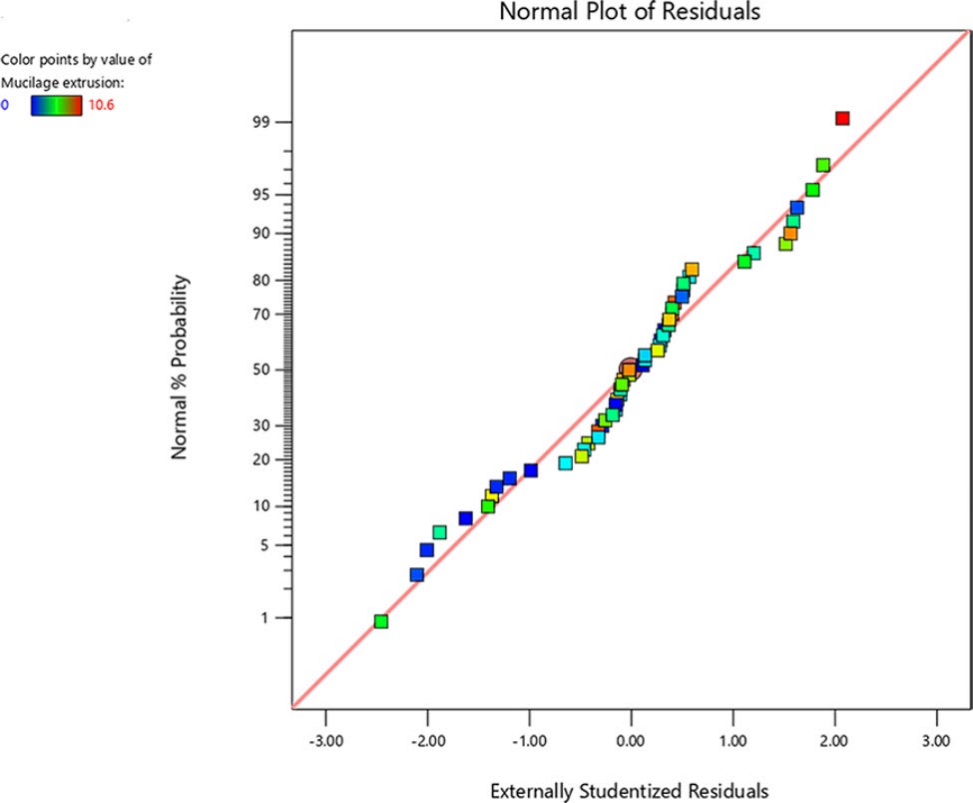
The normal probability plot compared to externally studentized residual plot of the mucilage secretion of *P. ovata, A. desertorum*, and *O. basilicum* seed

**Fig. 3:**
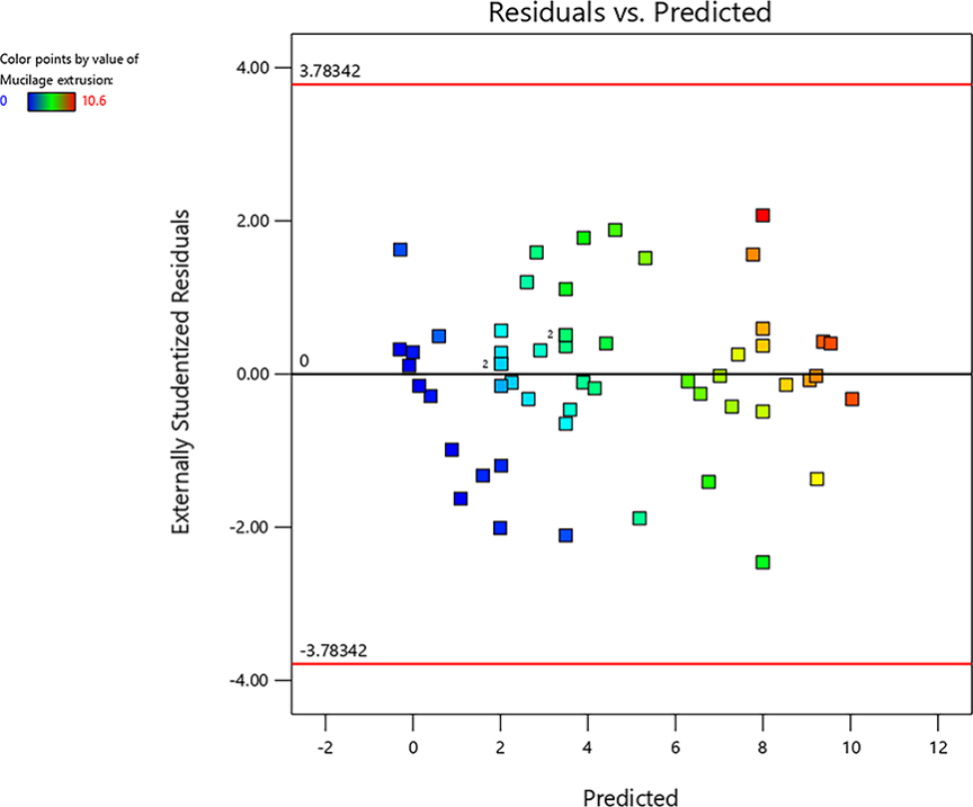
The externally studentized residual versus predicted value of the mucilage secretion of *P. ovata, A. desertorum*, and *O. basilicum* seed

Figure 2 shows the normal probability versus studentized residual plots, which indicate that the data distribution was normal. Moreover, Fig. 3 displays the externally studentized residual versus predicted values. Since the response values exhibit a random scatter plot and the original observations’ variance remains consistent, there is no requirement for transforming the response variables.

**Fig. 4:**
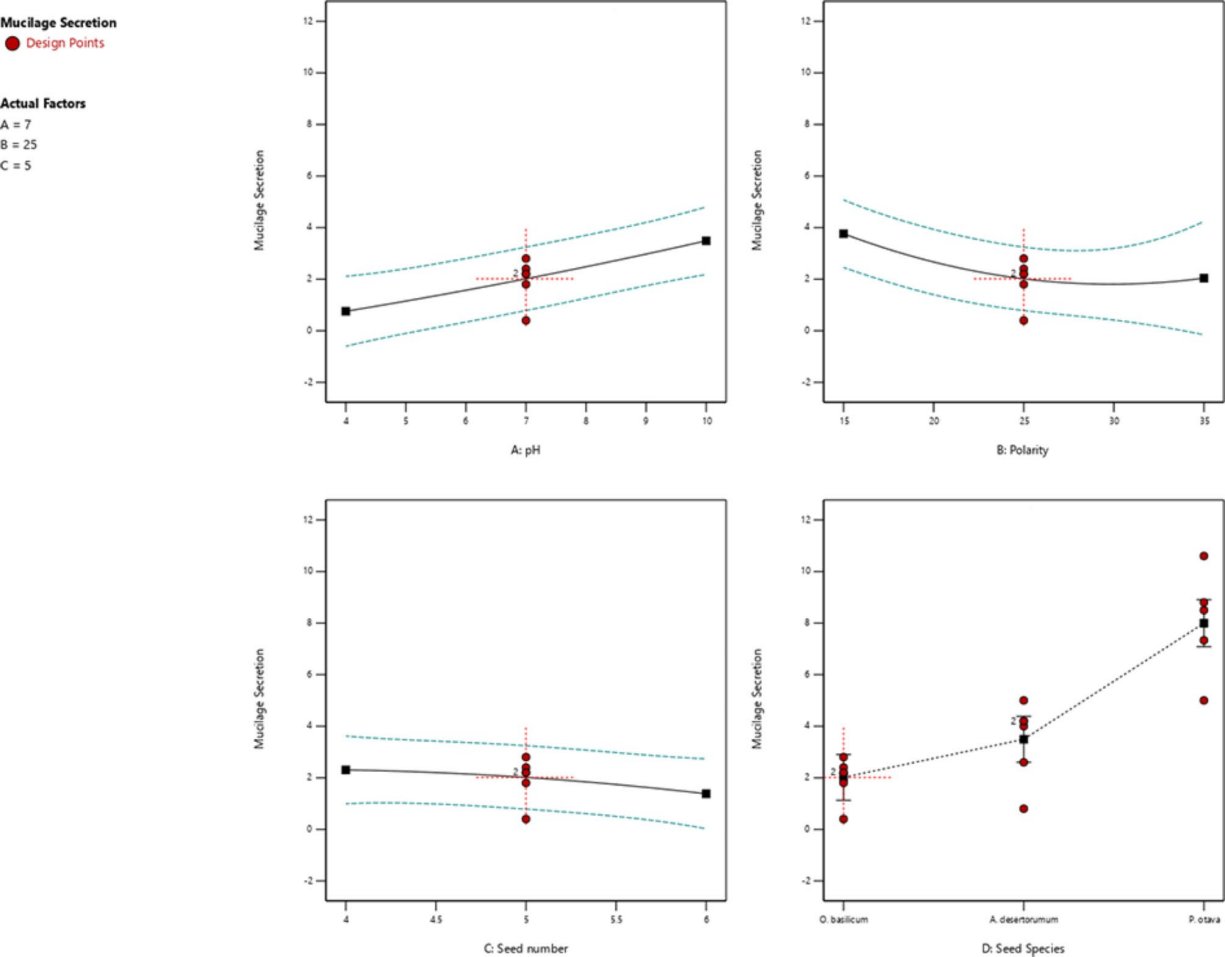
The major effect of (**a**) pH (X_1_), (**b**) Polarity (X_2_), (**c**) Seed number (X_3_), and (**d**) Species (X_4_) on the mucilage secretion

The ANOVA results (Table 3) allowed the effects of the experimental factors on mucilage secretion to be determined. Figure 4 depicts the corresponding two-dimensional response surface plots. Notably, pH, seed number, polarity, and species were determined to exert significant influences on mucilage secretion. The RSM design experiments demonstrated that these four factors influence mucilage secretion. As shown in Table 3, seed species (X_1_) was identified as having a substantial impact on mucilage secretion compared to the other variables, while seed number was found to have exerted relatively minor impacts on mucilage secretion.

**Fig. 5:**
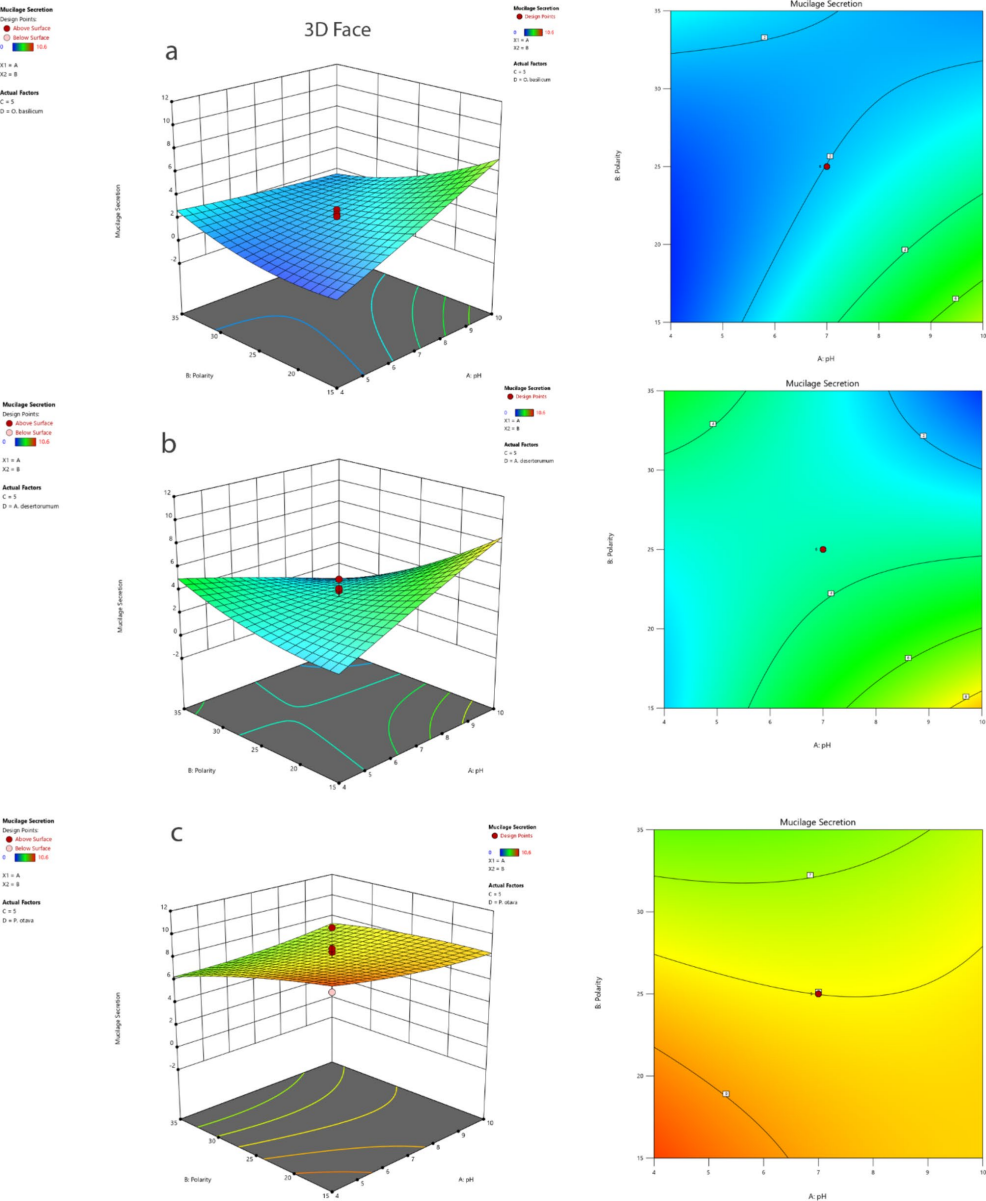
Interactions between (**a**) pH-Polarity (X_1_ × _2_) in *O. BASILICUM* (**b**) pH-Polarity (X_1_ × _2_) in *A. DESERTORUM* and (**c**) pH-Polarity (X_1_ × _2_) in *P. OVATA* at average seed number with 3D response surface plots and contour

Figure 5 depicted the interactions between variables. It indicates the interaction effect of pH (X_1_) and Polarity (X_2_) in a constant average seed number is the most effective interaction factor for mucilage secretion. The mucilage secretion of *O. basilicum* and *A. desertorum* increases with the increase of Polarity and decrease of pH (Fig. 5a and b). However, as illustrated in Fig. 5c, the mucilage secretion of *P. ovata* displays an increase with the decrease in both Polarity and pH.

**Fig. 6:**
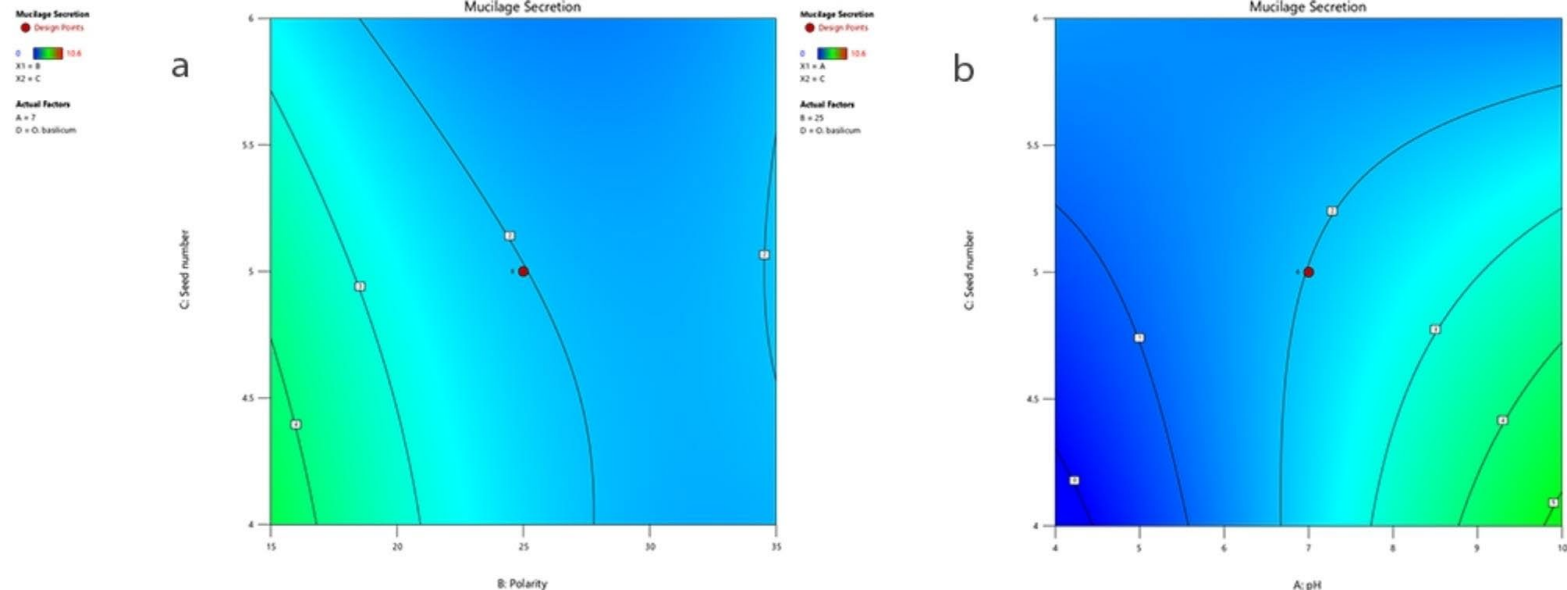
Interactions between (**a**) Polarity-Seed number (X_2_ × _3_) (**b**) pH-Seed number (X_1_ × _3_) in *O. BASILICUM* at average over of pH and seed species with with 3D response surface plots and contour

The interaction of pH (X1) and seed number (X3) on mucilage secretion, considering the average values of polarity and seed species, is presented in Fig. 6a. Additionally, the interaction effect between polarity (X2) and seed number (X3) on mucilage secretion, accounting for the average values of pH and seed species, as well as the visualization of the three-dimensional response surface, are depicted in Fig. 6b.These graphs indicate that these interactions have less significant effects on mucilage secretion.

According to the study, reducing the polarity in *P. ovata* and *A. desertorum* seeds leads to an increase in mucilage secretion. This effect is even more pronounced in *O. basilicum*, where the increase in mucilage secretion is around 25%, and after a slight decrease, it increases again up to 35%. More precisely, reducing the polarity from 35 to 15% in *O. basilicum*, *A. desertorum*, and *P. ovata* led to respective increases in mucilage secretion by factors of 1.87, 1.88, and 1.35. These findings suggest that reducing the polarity of seeds can be an effective way to increase mucilage secretion.

**Fig. 7:**
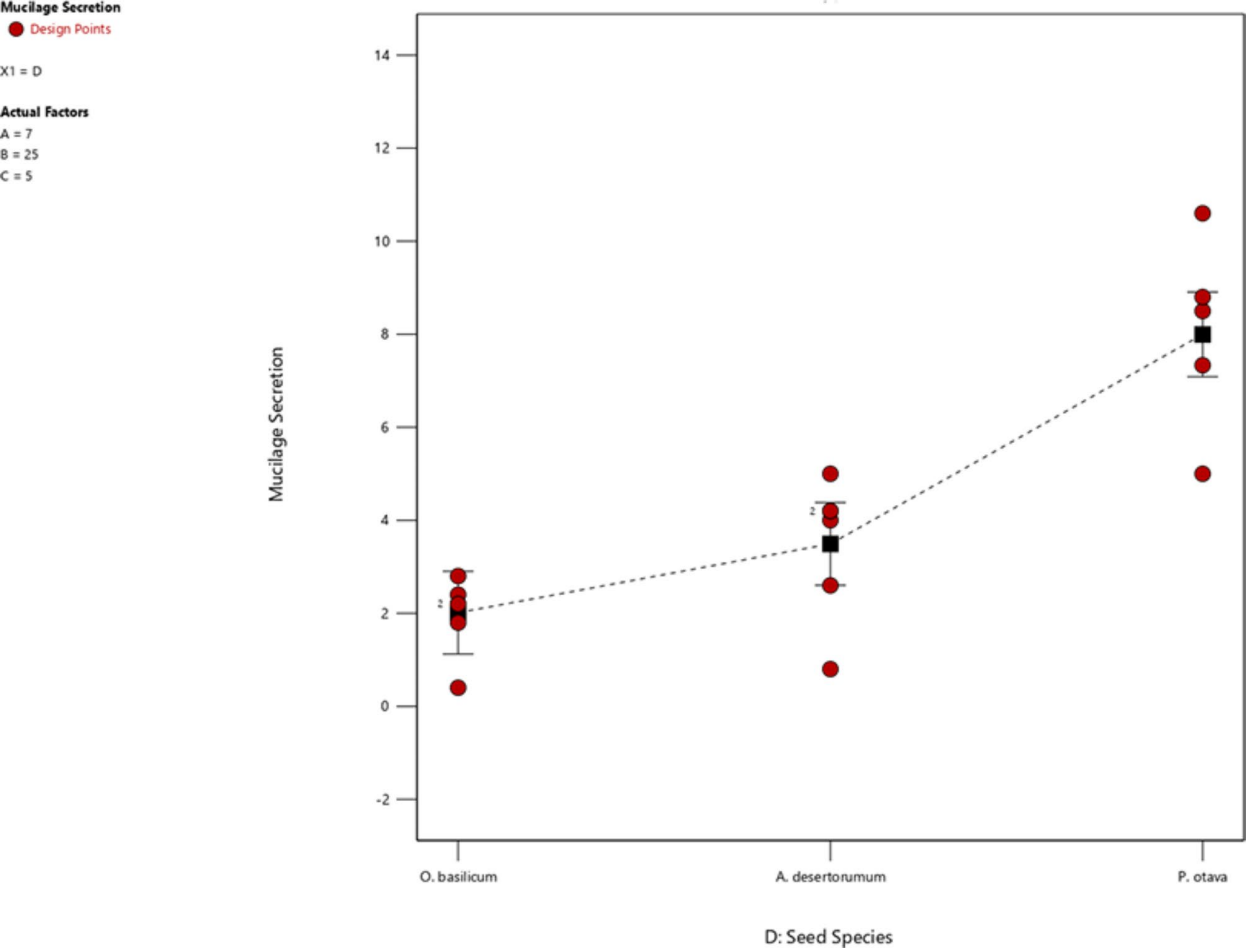
Effect of seed species on mucilage secretion of *P. ovata, A. desertorum*, and *O. basilicum* seed

Based on Table 4; Fig. 7, it can be observed that the amount of mucilage secretion in *O. basilicum* and *A. desertorum* seeds increases as the pH increases. Elevating the pH from 4 to 10 in *O. basilicum* and *A. desertorum* seeds resulted in mucilage amounts increasing by 4.6 and 1.33 times, respectively. However, in the case of *P. ovata* seed, the mucilage amount decreased to 0.97 when the pH was increased. These results suggest that controlling the pH levels can be an effective way to regulate mucilage secretion in different types of seeds.

**Table 4:** Effect of polarity on mucilage secretion of *P. ovata, A. desertorum*, and *O. basilicum* seed

*O. BASILICUM*	*A. DESERTORUM*	*P. OVATA*	pH and Polarity
0.75	2.87	8.4	pH = 4
2.01	3.49	7.99	pH = 7
3.49	3.83	8.18	pH = 10
3.76	5.53	8.84	Polarity = 15%
2.01	3.49	7.99	Polarity = 25%
2.04	2.94	6.51	Polarity = 35%

### Optimization by response surface modeling

The primary objective of the experimental study was to identify the optimal conditions that lead to the highest possible mucilage secretion. Based on the results, the optimum operating conditions for mucilage secretion were found to be 10.3 at polarity = 15 and pH = 4 for *P. ovata*, 9.4 at polarity = 15 and pH = 10 for *O. basilicum*, and 9.2 at polarity = 15 and pH = 10 for *A. desertorum* (as shown in Table 5). The optimal pH for mucilage secretion can vary depending on the plant source and the properties of the mucilage [14, 15]. According to the literature, at high pH, the extraction of mucilage can be enhanced because the alkaline conditions can help to facilitate the release of mucilage [8, 14, 16]. Therefore, the increased secretion of mucilage in *O. basilicum* and *A. desertorum* seeds may be due to alkaline conditions. However, the choice of secretion solvent also depends on the properties of the mucilage. For example, some mucilage compounds may be sensitive to high-polarity solvents, which can cause degradation or changes in their functional properties. Therefore, optimization of the secretion solvent depends on the type of seeds [17–19]. These findings suggest that the optimization of pH and polarity levels can be an effective way to maximize mucilage secretion in different types of seeds.

**Table 5:** Optimal suggested points for variable levels to achieve the maximum mucilage secretion

pH	polarity	Seed Spices	Mucilage secretion	Desirability
4.0	15.000	*P. ovata*	10.338	0.975
10.0	15.000	*O. basilicum*	9.358	0.883
10.0	15.000	* A. desertorum*	9.222	0.870

To validate the model, a set of 9 experiments encompassed all three significant variables considered in the study. The experimental data on mucilage secretion from these trials were observed to align with the statistically predicted data, with a deviation of less than 5% from the predicted error.Based on these findings (as shown in Table 6), the authenticity of the model was confirmed. The validation of the model through experimental data suggests that the developed model can be reliably used to predict mucilage secretion in different types of seeds under various conditions. In summary, the outcomes of the response surface analyses have validated the notable influence of pH, polarity, and seed species on the process of mucilage secretion.

**Table 6:** Validation of the optimized model

Mucilage secretion	Predicted Mean	SD	SE	95% low	Mean	95% high
*P. ovata*	10.3	1.4	1.4	7.4	10.1	13.2
*O. basilicum*	9.3	1.4	1.5	6.1	9.2	12.6
* A. desertorum*	9.2	1.4	1.4	6.2	9.0	12.2

## Conclusion

The aim of this research was to determine the optimal conditions for mucilage secretion and investigate the holding capacity conditions for mucilage seeds of *P. ovata, O. basilicum*, and *A. desertorum*. This study investigated the impact of species, polarity, pH and seed number on mucilage secretion. The results indicated that the number of seeds had the least effect on mucilage secretion, while the species of seed had the most significant effect. Overall, the RSM design experiments confirmed the substantial influence of pH, seed species, and polarity on the process of the secretion of mucilage. The conditions yielding optimal secretion of 10.3 mm of *P. ovata* mucilage, 9.4 mm of *O. basilicum* mucilage, and 9.2 mm of *A. desertorum* mucilage were found to be pH = 4 and polarity = 15, pH = 10 and polarity = 15, and pH = 10 and polarity = 15, respectively. Based on the optimal conditions, it appears that a polarity of 15 is suitable for achieving the maximum amount of mucilage secretion in all three seeds. However, wheat and barley seeds had the highest amount of mucilage secretion in an alkaline environment, while basil seeds had the highest amount of mucilage secretion in an acidic environment. This result can serve as a useful guide for seed selection based on soil conditions. It appears that under suitable conditions, mucilage not only retains water for the seed but can also improve the seed’s conditions based on soil properties. Mucilage generally functions as an adaptation factor against osmotic stress, drought, or salinity during germination.

### Limitation

The findings of this study have to be seen in light of some limitations.

- The first limitation due to Iran’s sanctions, access to some articles and sources was not possible.
- The small size of the seeds and the mucilage around them caused limitations in maintaining the accuracy of the measurement.
- Due to the limited budget, there was a limitation in the supply of some raw materials, equipment, and technicians to conduct tests.
- Due to the one-year time limit of the project, the effect of a limited number of variables on the amount of mucilage production was investigated. It is necessary to examine more variables in future research.
- In this research, a scale with a readability of 0.0001 was used for measurement because a scale with more accuracy was not available.

## Data Availability

Data supporting of this research are available upon reasonable request from the corresponding author.

## References

[1] Yang X, Baskin JM, Baskin CC, Huang Z. “More than just a coating: Ecological importance, taxonomic occurrence and phylogenetic relationships of seed coat mucilage,” *Perspect. Plant Ecol. Evol. Syst*, vol. 14, no. 6, pp. 434–442, Dec. 2012, 10.1016/j.ppees.2012.09.002.

[2] Geneve RL, Hildebrand DF, Phillips TD, Al-Amery M, Kester ST. Stress influences seed germination in mucilage-producing chia. Crop Sci. 2017;57(4):2160–9. 10.2135/cropsci2016.08.0703.

[3] Brütsch L, Stringer FJ, Kuster S, Windhab EJ, Fischer P. Chia seed mucilage - A vegan thickener: isolation, tailoring viscoelasticity and rehydration. Food Funct. 2019;10(8):4854–60. 10.1039/c8fo00173a. 31328195 10.1039/c8fo00173a

[4] Di Marsico A, et al. Mucilage from fruits/seeds of chia (Salvia hispanica L.) improves soil aggregate stability. Plant Soil. 2018;425:1–2. 10.1007/s11104-018-3565-1.

[5] Šola K, Dean GH, Haughn GW. *Arabidopsis seed mucilage: A specialised extracellular matrix that demonstrates the structure-function versatility of cell wall polysaccharides*, vol. 2, no. 4. 2019.

[6] Puligundla P, Lim S. A review of extraction techniques and food applications of Flaxseed Mucilage. Foods. 2022;11(12). 10.3390/foods11121677. 10.3390/foods11121677 PMC9223220 35741874

[7] Engelbrecht M, Bochet E, García-Fayos P. Mucilage secretion: an adaptive mechanism to reduce seed removal by soil erosion? Biol J Linn Soc. 2014;111(2):241–51. 10.1111/bij.12198.

[8] Liu Y, Hu X, Ye Y, Wang M, Wang J. “Emulsifying properties of wheat germ:Influence of pH and NaCl,” *Food Hydrocoll*, vol. 100, no. August 2019, p. 105431, 2020, 10.1016/j.foodhyd.2019.105431.

[9] Yılmaz C, Gökmen V. Kinetic evaluation of the formation of tryptophan derivatives in the kynurenine pathway during wort fermentation using Saccharomyces pastorianus and Saccharomyces cerevisiae. Food Chem. 2019;297:124975. 10.1016/j.foodchem.2019.124975. 31253324 10.1016/j.foodchem.2019.124975

[10] Ren L, et al. Physicochemical, Rheological, Structural, antioxidant, and Antimicrobial Properties of Polysaccharides extracted from tamarind seeds. J Food Qual. 2022;2022. 10.1155/2022/9788248.

[11] Cherrate M, et al. Effects of temperature, pH, and salinity on seed germination of Acinos alpinus subsp. Meridionalis and FTIR Analysis of Molecular Composition Changes. Sustainability. 2023;15(6):4793. 10.3390/su15064793.

[12] Mannai F, Elhleli H, Yılmaz M, Khiari R, Belgacem MN, Moussaoui Y. Precipitation solvents effect on the extraction of mucilaginous polysaccharides from Opuntia ficus-indica (Cactaceae): Structural, functional and rheological properties. Ind Crops Prod. 2023;202:117072. 10.1016/j.indcrop.2023.117072.

[13] Ziolkovska A. Laws of flaxseed mucilage extraction. Food Hydrocoll. 2012;26(1):197–204. 10.1016/j.foodhyd.2011.04.022.

[14] Javed MT, Stoltz E, Lindberg S, Greger M. Changes in pH and organic acids in mucilage of Eriophorum angustifolium roots after exposure to elevated concentrations of toxic elements. Environ Sci Pollut Res. 2013;20(3):1876–80. 10.1007/s11356-012-1413-z. 10.1007/s11356-012-1413-z 23274805

[15] Hoag S, Peng X, Ogaji I, Okafor I. Effect of pH on the viscosity of grewia mucilage. Chronicles Young Sci. 2012;3(2):141. 10.4103/2229-5186.98687.

[16] Knott M, Ani M, Kroener E, Diehl D. Effect of changing chemical environment on physical properties of maize root mucilage. Plant Soil. 2022;478:1–2. 10.1007/s11104-022-05577-0.

[17] Rambwawasvika H, Parekh CT, Naidoo B, Chiririwa H. “Extraction and Characterisation of Mucilage from the herb Dicerocaryum senecioides and its use a potential hair permanent,” *Int. J. Appl. Chem*, vol. 13, no. 3, pp. 691–705, 2017, [Online]. Available: http://www.ripublication.com.

[18] Oh S, Kim DY. Characterization, antioxidant activities, and Functional Properties of Mucilage extracted from Corchorus olitorius L. Polym (Basel). 2022;14(12). 10.3390/polym14122488. 10.3390/polym14122488 PMC9228403 35746064

[19] Quintero-García M, et al. Comparative analysis of the chemical composition and physicochemical properties of the mucilage extracted from fresh and dehydrated opuntia ficus indica cladodes. Foods. 2021;10(9):1–18. 10.3390/foods10092137. 10.3390/foods10092137 PMC8471229 34574247

